# Umbilical Cord Teratoma Presenting as Ruptured Omphalocele

**DOI:** 10.1055/s-0041-1741509

**Published:** 2022-02-03

**Authors:** Fabian Doktor, Jan-Hendrik Gosemann, Peter Zimmermann, Manuela Siekmeyer, Holger Stepan, Martin Lacher

**Affiliations:** 1 Department of Pediatric Surgery, University of Leipzig, Leipzig, Saxony, Germany; 2 Department of Pediatrics, University of Leipzig, Leipzig, Saxony, Germany; 3 Division of Obstetrics, University of Leipzig, Leipzig, Saxony, Germany; 4 Klinik und Poliklinik für Kinderchirurgie, Universitätsklinikum Leipzig, Leipzig, Germany

**Keywords:** umbilical cord teratoma, omphalocele, abdominal wall defect

## Abstract

Congenital mature teratomas of the umbilical cord are extremely rare. We report on a girl who presented with a ruptured omphalocele and a 7 cm mass connected to the umbilicus, which we resected on the first day of life. Histology revealed mature umbilical cord teratoma . On the 29th day of life, a secondary laparotomy was necessary to address the associated intestinal malformations (megaduodenum, stenotic small bowel with duplication and malrotation). After a prolonged hospital stay, we discharged the patient in age-appropriate conditions. Antenatal diagnosis of an umbilical cord tumor can be challenging in the presence of an omphalocele. Given the high prevalence of associated malformations, the finding of umbilical cord teratoma should be followed by a detailed and comprehensive neonatal workup for additional abnormalities.

## Introduction

Teratomas can be subgrouped into extragonadal and gonadal. Two-thirds of these tumors are extragonadal and one-third is of gonadal origin. 1 Sacrococcygeal teratoma is the most frequent subtype of extragonadal teratoma in childhood. 2 3 Umbilical cord teratoma (UCT) was first described in 1878 by Budin in a full-term female newborn with a mass as big as an “adults' fist.” 4 We present a female neonate with a ruptured omphalocele and an umbilical mass which turned out to be an UCT.

## Case Report

A non-smoking 34-year-old, first gravida, was referred to our hospital due to an antenatal ultrasound finding suggesting gastroschisis. The past medical history revealed osteogenesis imperfecta of the child's mother and maternal grandmother. After 36 + 6 weeks of gestation, a preterm newborn of 2,735 g (30th percentile) was delivered via C-section.

**Fig. 1:**
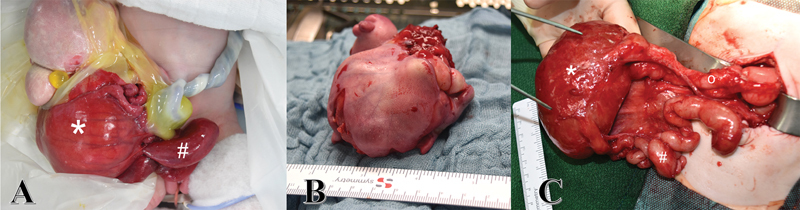
( **A** ) Ruptured omphalocele with umbilical cord teratoma (size of 7cm) at birth and ( **B** ) after resection. ( **C** ) Proximal duodenum (o) leading into the distal cystic duodenum (*) with a caliber change of 6:1 followed by proximal stenotic jejunum (#).

At birth a ruptured omphalocele with a large cystic, solid tumor connected to the umbilicus was seen ( Fig. 1A ). The mass was partially covered with skin and attached to the small bowel. We took the child to the operating room for explorative laparotomy on the first day of life.

During this procedure, the mass was carefully separated from the adherent small bowel and resected ( Fig. 1B ). The duodenum and jejunum were patent, but the second part of the duodenum and the proximal jejunum is massively enlarged with a caliber change of 6:1 ( Fig. 1C ). The remaining 40 cm of the small bowel was of small diameter, filled with meconium and malrotated. As the megaduodenum was semicircumferentially surrounded by pancreas and the Vater's papilla leading into it, an extensive reconstructive procedure on the duodenum was not felt to be the best option. Instead, we decided to close the abdomen at this point to opt for revisional surgery.

On histology, the resected mass contained skin, respiratory epithelia, muscle tissue, adipose tissue, bone and cartilage without immature elements corresponding to a mature teratoma (G0 Gonzalez-Crussi). 5 Alpha-fetoprotein (AFP) and beta human chorionic gonadotropin (β-hCG) were not elevated at the time of diagnosis. During the prolonged hospital stay of 8 months, it took another three laparotomies to deal with all intestinal malformations and establish bowel function.

**Fig. 2:**
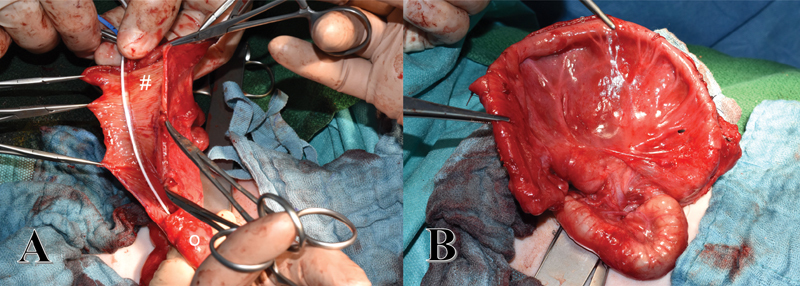
( **A** ) First revisional laparotomy on the 29th day of life. Opening of the megaduodenum, nasogastric tube is bridging the lumen to the following small bowel. Second part of the duodenum after tapering (#). Distal duodenum (o). ( **B** ) Reconstructed duodenum and proximal jejunum after tapering.

Upper gastrointestinal series were taken on the 29th day of life. These showed an obstruction at the level of the duodenum. During the following relaparotomy, the megaduodenum was tubularized ( Fig. 2A and B ). Two masses adherent to the head of the pancreas were not resected at this point. A third laparotomy on the 67th day of life was necessary due to a functional stenosis between the tubularized duodenum and the jejunum. We decided to resect the stenosis by creating a proximal diamond-shaped duodenojejunostomy and a distal jejunojejunostomy. In addition, a gastrostomy was placed. The 4th laparotomy on the 134th day of life was necessary to remove the two masses close to the pancreas (2 and 1.5 cm in diameter on magnetic resonance imaging [MRI). Histology again revealed mature teratoma.

At this time, the length of the small bowel was 80 cm. Parenteral nutrition was given until 5 months of age. After 8 months, the patient was discharged from the hospital on full enteral feeds. After a follow-up of 23 months, the girl has no developmental deficits and no signs of a tumor recurrence on MRI or elevated tumor markers (AFP or β-hCG).

## Discussion

Teratoma of the umbilical cord is extremely rare. At present, there are only 18 other cases reported. 6 7 In UCT, there is a female predominance with 14 female, two male, and two cases of unknown gender.

A teratoma derives from more than one germ cell layer. Most of UCTs comprise mature elements comparable to our case. 8 9 Only four immature teratomas of the umbilical cord have been described with an outcome comparable to the mature teratomas. 6 10 11 12 Today, the diagnosis of an UCT is frequently confirmed antenatally with the earliest detection at 13 weeks of pregnancy. 6 However, in our case, the antenatal ultrasound did not consider a teratoma but instead gastroschisis.

UCT can reach significant sizes. The mean diameter of all described UCT is 8.8 cm. 6 This corresponds to the case presented here with a maximum size of 7 cm in diameter. The biggest UCT has been reported by Crahes et al, 11 who encountered a tumor with a maximum diameter of 23 cm. Therefore, potential complications comparable to sacrococcygeal teratoma such as fetal hydrops, high output cardiac failure, and massive bleeding from the cord vessels may ultimately lead to intrauterine demise. 6

Associated malformations are frequently described as nine of the 18 other cases had multiple malformations. 4 6 8 9 13 14 15 16

Like in our child, the most prevalent additional anomaly is an abdominal wall defect in 46% of cases mostly omphalocele. 10 11 12 17 18 19 20 One of the reported UCT was seen in a child with trisomy 13. 19 Thirteen percent of the newborns with UCT and associated malformations show dysmorphic intestine. Our patient underwent multiple laparotomies for a megaduodenum, an atresia-like hypoplastic small bowel and resection of teratomatous remnants. The complete removal during the first surgery was unsuccessful because of the proximity of the teratoma to the small bowel and the pancreas. In the literature, two other neonates are reported with intestinal atresia 10 and dilation of the small bowel. 11 Moreover, 13% of the reported cases had urinary malformations, for example, bladder extrophy or kidney aplasia 21 22 and 26% were diagnosed with other malformations in different parts of the body (hydrocephalus, limb deformities, meningomyelocele, atrioventricular canal defect). 11 21 22

At the age of 2 years, our patient is thriving and developing appropriately to her age. There is neither recurrent tumor on MRI nor indicated by serologic markers. In 8 of the 18 other reported cases (44%), the outcome was favorable without developmental deficits. Conversely, eight of the newborns (44%) did not survive the first year of life. Among those were three patients of intrauterine demise, 9 17 19 four neonatal deaths within the first month, 10 16 20 22 and one child who died in the first 12 months. 6 The outcome of the two remaining patients (11%) was not reported.

## Conclusion

If prenatal imaging suggests an abdominal wall defect especially in a female fetus, the rare differential diagnosis of an UCT should be taken into consideration and associated malformations expected. As seen in our case, the surgical treatment may be complex.

## Notes

New Insights & the Importance for the Pediatric Surgeon An umbilical cord teratoma is a spot diagnosis. Every pediatrician and pediatric surgeon should be aware of possible associated malformations.
